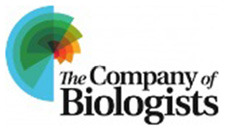# Sex Differences in Aggression Are Paralleled by Differential Activation of the Brain Social Decision-Making Network in Zebrafish

**DOI:** 10.3389/fnbeh.2022.784835

**Published:** 2022-02-16

**Authors:** María Florencia Scaia, Ibukun Akinrinade, Giovanni Petri, Rui F. Oliveira

**Affiliations:** ^1^Instituto de Biodiversidad y Biología Experimental y Aplicada—CONICET, Ciudad Auntónoma de Buenos Aires, Buenos Aires, Argentina; ^2^Laboratorio de Neuroendocrinología y Comportamiento, Departamento de Biodiversidad y Biología Experimental, Facultad de Ciencias Exactas y Naturales, Universidad de Buenos Aires, Ciudad Autónoma de Buenos Aires, Buenos Aires, Argentina; ^3^Instituto Gulbenkian de Ciência, Oeiras, Portugal; ^4^Hotchkiss Brain Institute and the Department of Physiology and Pharmacology, Cumming School of Medicine, University of Calgary, Calgary, AB, Canada; ^5^ISI Foundation and ISI Global Science Foundation, Torino, Italy; ^6^ISPA– Instituto Universitário, Lisbon, Portugal; ^7^Champalimaud Neuroscience Programme, Lisbon, Portugal

**Keywords:** aggressive behavior, sex-differences, social decision-making network, contest, fish

## Abstract

Although aggression is more prevalent in males, females also express aggressive behaviors and in specific ecological contexts females can be more aggressive than males. The aim of this work is to assess sex differences in aggression and to characterize the patterns of neuronal activation of the social-decision making network (SDMN) in response to intra-sexual aggression in both male and female zebrafish. Adult fish were exposed to social interaction with a same-sex opponent and all behavioral displays, latency, and time of resolution were quantified. After conflict resolution, brains were sampled and sex differences on functional connectivity throughout the SDMN were assessed by immunofluorescence of the neuronal activation marker pS6. Results suggest that both sexes share a similar level of motivation for aggression, but female encounters show shorter conflict resolution and a preferential use of antiparallel displays instead of overt aggression, showing a reduction of putative maladaptive effects. Although there are no sex differences in the neuronal activation in any individual brain area from the SDMN, agonistic interactions increased neuronal activity in most brain areas in both sexes. Functional connectivity was assessed using bootstrapped adjacency matrices that capture the co-activation of the SDMN nodes. Male winners increased the overall excitation and showed no changes in inhibition across the SDMN, whereas female winners and both male and female losers showed a decrease in both excitation and inhibition of the SDMN in comparison to non-interacting control fish. Moreover, network centrality analysis revealed both shared hubs, as well as sex-specific hubs, between the sexes for each social condition in the SDMN. In summary, a distinct neural activation pattern associated with social experience during fights was found for each sex, suggesting a sex-specific differential activation of the social brain as a consequence of social experience. Overall, our study adds insights into sex differences in agonistic behavior and on the neuronal architecture of intrasexual aggression in zebrafish.

## Introduction

According to sexual selection theory in most species, sex differences in fecundity lead to differential selection regimes between the sexes such that the fitness of males is dependent on the number of matings, whereas the fitness of females is dependent on the quality of matings (Anderson, 1994). As a consequence, typically males compete for mating opportunities that allow them to increase their mating frequency, whereas females are less competitive and more selective when choosing mates (Darwin, 1871; Anderson, 1994). This increased intrasexual competition among males over mating opportunities has been seen as the major selective force for the evolution of a male skew in aggressiveness, accompanied by weaponry (e.g., canine teeth, horns) and large body size in males (Lindenfors and Tullberg, 2011). However, aggressiveness is also expressed in other ecological contexts, such as competition for food, or brood defense. Therefore, although aggression is more prevalent in males, it is also expressed by females, which can be more aggressive than males in specific ecological contexts (Oliveira and Almada, 1996; Borg et al., 2012; Renn et al., 2012; Scaia et al., 2018a, b). Thus, male and female aggression can be functionally different, with female aggression usually related, but not restricted, to maternal care at specific reproductive stages. For example, in African cichlid fish, while males display intrasexual territorial aggression, females become aggressive when brooding and defending fry against intruders (*Astatotilapia burtoni*: Renn et al., 2009; Maruska and Fernald, 2010; *Oreochromis mossambicus*: Oliveira and Almada, 1996). On the other hand, both male and female aggression can serve similar ecological functions, as in the case of joint defense of breeding territories in monogamous cichlids with biparental care (e.g., *Cichlasoma dimerus*: Tubert et al., 2012; Scaia et al., 2020). In such cases, female aggressive behavior during staged agonistic encounters in neutral aquaria is as high as that of males, even in the absence of the mate or brood, suggesting that within the same functional context, female aggression is motivated by similar cues as in males. Thus, female aggression can be a non-adaptive by-product that reflects a correlated evolutionary response to selection acting on males, or it can have been directly selected (Rosvall, 2013).

Characterizing the molecular pathways and neuronal circuits underlying aggressive behavior is critical to determine the extent to which proximate mechanisms of aggression are similar between the sexes. The Social Decision-Making Network (SDMN) is an evolutionarily conserved brain network across vertebrates regulating social behaviors, including aggression, such that the expression of behavior can be better explained by the overall pattern of network activity rather than by the activity at a single node (Goodson, 2005; O’Connell and Hofmann, 2011; Teles et al., 2015). Moreover, most nodes of the SDMN express receptors for neuromodulators (e.g., vasotocin and oxytocin) and peripheral hormones (sex steroids and glucocorticoids), which allow this network to be modulated by the internal state and life-history stage of the organism (O’Connell and Hofmann, 2011). Thus, the SDMN is an obvious target to assess to what extent male and female aggression share the same mechanisms. The occurrence of shared mechanisms would support a common evolutionary origin or a response to similar selection pressures, of male and female aggression, whereas the occurrence of different mechanisms would fit better a scenario of different evolutionary origins, putatively under different selective pressures, for male and female aggression (Rosvall, 2013).

Zebrafish (*Danio rerio*) is an established model in behavioral neuroscience and, since both males and females show territorial aggression (Spence et al., 2008; Filby et al., 2010; Paull et al., 2010), it provides an excellent opportunity for testing the hypothesis related to sex differences in the neural mechanisms underlying aggressive behavior (Filby et al., 2010; Teles et al., 2013, 2016a,b; Teles and Oliveira, 2016). In fact, both males and females establish dominance hierarchies (Paull et al., 2010), and aggression is used by males to defend mating territories (Spence and Smith, 2005) and by both sexes during foraging, where dominant individuals try to monopolize food sources (Grant and Kramer, 1992; Hamilton and Dill, 2002; Spence et al., 2008).

The aim of the present work is to assess sex differences in aggression and to characterize the patterns of neuronal activation of the social-decision making network that respond to agonistic encounters in both males and females. The phospho-S6 ribosomal protein (pS6) has been used as a molecular marker of neuronal activity since it has been shown that it becomes phosphorylated in activated neurons (Knight et al., 2012). This neuronal activity marker has a good colocalization with other activity markers (e.g., *c-fos*; Knight et al., 2012) and has already been used successfully in other studies with fish (e.g., Butler et al., 2018; Maruska et al., 2020). Based on the number of pS6 positive cells in different regions of the SDMN we tested if winning or losing an agonistic interaction elicited similar patterns of activation in each brain region that is part of the SDMN between the sexes and if there are sex differences in the network structure of the SDMN, which captures functional connectivity across the SDMN (i.e., excitation-inhibition balance and network hubs).

## Materials and Methods

### Animals

We used adult wild-type (AB) zebrafish (60 males and 60 females) bred and held at Instituto Gulbenkian de Ciência (IGC, Oeiras, Portugal). Fish were kept in a recirculating system (ZebraTec, 93 Tecniplast), at 28°C with a photoperiod of 14 light (7 a.m. to 9 p.m.) : 10 darkness (9 p.m. to 7 a.m.) in mixed tanks until 3 months of age. The water system was monitored for nitrites (<0.2 ppm), nitrates (<50 ppm), and ammonia (0.01–0.1 ppm). Conductivity and pH were maintained at 700 μS cm^−1^ and 7, respectively. Fish were fed twice a day with commercial food flakes (Bionautic) and *Artemia salina*, except on experimental days.

### Experimental Protocol

**Figure 1:**
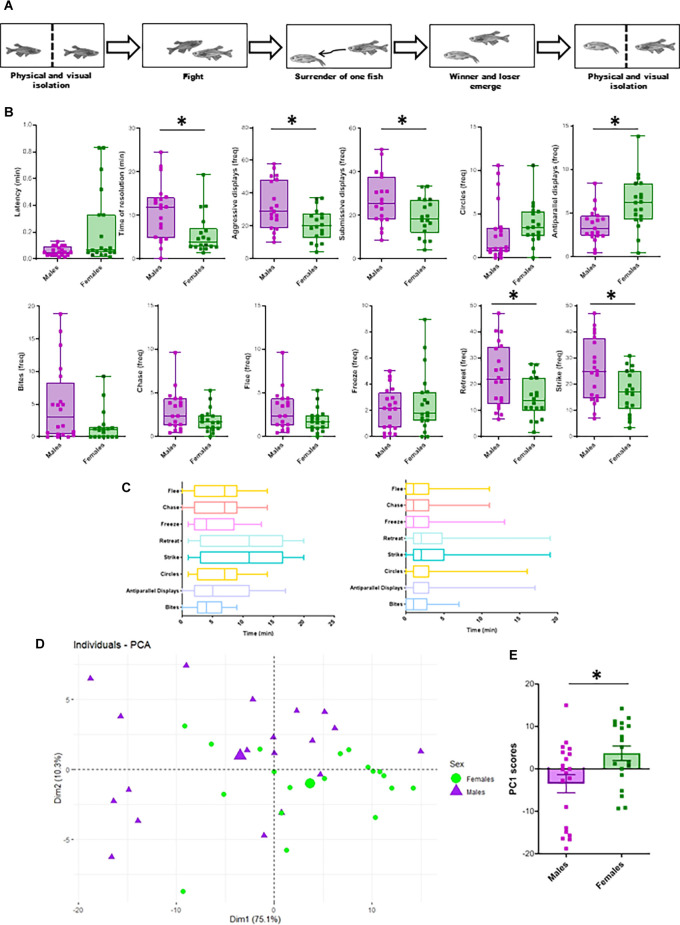
Sex differences in aggressive behavior. **(A)** Schematic illustration of experimental protocol for zebrafish fighting. After overnight physical and visual isolation, sex-matched dyads perform agonistic behaviors until one of the fish surrenders. After winner and loser determination, both opponents were isolated again until tissue collection. **(B)** Sex differences in behavioral parameters. Latency and time of resolution are expressed in minutes. The frequency of each agonistic behavior is calculated as the number of each behavioral display, divided by the duration of the encounter until conflict resolution. Aggressive behaviors include antiparallel displays, circles, bites, chases and strikes, while submissive behaviors include freeze, flee, and retreats (Oliveira et al., 2011). Box plots were used to plot the data: the box extends to the furthest data points within the 25th and 75th percentile, and whiskers extend to the furthest data points not considered outliers. Asterisks indicate significant differences (*p* < 0.05) using *t*-test (frequency of aggressive and submissive behaviors, antiparallel displays, chases, flee, strikes, retreats) or Mann-Whitney (latency, time of resolution, frequency of bites, circles, freeze). **(C)** Temporal dynamics of male-male (left) and female-female (right) encounters. All behaviors were analyzed and quantified during each dyadic encounter, and graphs indicate the presence of each behavior throughout all encounters. Error bars represent the earliest and latest moment in which each behavior is observed. The line inside the boxes represenst the median. **(D)** PCA-based on all behavioral traits (bites, circles, chase, antiparallel displays, flee, strike, retreat, and freeze) in males and females. Purple triangles indicate male individual scores and green dots indicate female individual scores for PC1 and PC2, whereas the larger purple triangle indicates the centroid of the male distribution and the larger green dot the centroid of the female data points Differences between the sexes were assessed using Euclidean dissimilarities and each test was conducted using 999 permutations of appropriate units (perMANOVA *p*-value = 0.012, *F* = 5.5502). **(E)** Box plots comparing male and female PC1 scores (Mann-Whitney *U* test: *W* = 463.5, *p* = 0.0190).

A total of 20 female-female and 20 male-male dyadic agonistic encounters were analyzed for this study. A behavioral paradigm previously used to study agonistic interactions (Figure 1A, Oliveira et al., 2011; Teles et al., 2013) was followed. Briefly, animals were paired in sex and size-matched dyads and placed in an experimental arena (20 × 12.5 × 14.5 cm; length × width × height), which was divided into two compartments by a removable opaque partition. Members of each dyad were kept overnight in visual isolation, each in one compartment of the experimental arena. On the next day, the partition was removed and the fish were allowed to interact until conflict resolution, which was defined when a winner and a loser emerged and an asymmetry of expressed behaviors was observed for 2 min (i.e., when all aggressive acts were initiated by one fish, the dominant or winner), and the other fish, subordinate or loser, only displayed submissive behavior; Oliveira et al., 2011). Once this behavioral asymmetry was observed, winners and losers were identified and isolated by placing back the partition. As a consequence, this social treatment generated two behavioral states: winners (W, *n* = 20 per sex) and losers (L, *n* = 20 per sex). Non-interacting fish were used as control (i.e., visual and physical isolation, I, *n* = 10 per sex). All animals were tested in pairs in order to give them access to conspecific odors, which would otherwise only be present in fighting dyads, therefore avoiding confounding effects of putative chemical cues in the comparisons between treatments. Behavioral interactions were video-recorded with a JVC HD Everio camera (GZ-MS215) for subsequent behavioral analysis. The experiments were performed between 10:00 and 12:00 h to control for possible circadian variation in agonistic behavior.

### Behavioral Analysis

For the behavioral data, video recordings were analyzed by a blind observer and behavioral displays were distinguished following the ethogram for male zebrafish agonistic behavior (Oliveira et al., 2011). The following behavioral variables were quantified: (1) latency for the first attack (i.e., the time between the beginning of the recording period and the first aggressive behavior); (2) fight resolution time (i.e., the time needed for a behavioral asymmetry to be observed, this way allowing a social hierarchy to be established); (3) frequency of all behavioral traits (i.e., display, circle, bite, chase, strike, flee, and freeze); (4) frequency of aggressive traits; and (5) frequency of submissive behaviors during the social encounter. Aggressive behaviors included bite, chase, and strike, while submissive behaviors included freeze, flee and retreat (Oliveira et al., 2011).

### Specimen Processing and Tissue Collection

Immediately after conflict resolution was observed, social interactions were stopped by placing an opaque partition in each tank that separates the two opponents from each other. Fish remained in isolation in each of these compartments for 45 min after which they were euthanized with an overdose of tricaine solution (MS222, Pharmaq; 500–1,000 mg/L), followed by decapitation. The choice of 45 min post-interaction for the brain sampling is justified by the fact that this study focuses on the expression of the phospho-S6 ribosomal protein (pS6) neural activation marker, which has a time course of expression compatible with this sampling point. Previous research in mice and cichlid fish indicates an efficient time for ps6 expression to vary between 30 min or 1 h (Knight et al., 2012; Butler et al., 2018; Maruska et al., 2020), and an unpublished pilot study in our lab found no differences on the number of pS6 positive cells when comparing a post-exposure time course of 30, 45 and 60 min. Heads were collected and fixed in 10% formalin for 72 h, followed by decalcification in EDTA (0.5 M, pH 8) for 48 h. Samples were embedded in paraffin and coronal serial sections were cut at 5 μm, deparaffinized, hydrated, and used for subsequent immunofluorescence staining. Regions of interest corresponded to brain areas involved in the social decision-making network (O’Connell and Hofmann, 2011) and were identified according to their anatomical position following a zebrafish brain atlas (Wullimann et al., 1996). The following regions were identified: medial (Dm_m_), lateral (Dm_l_), and posterior portion (Dm) of the medial zone of Dorsal Telencephalic area, lateral zone of Dorsal Telencephalic area (Dl), rostral (Vv_r_) and caudal portion (Vv_c_) of the ventral nucleus of Ventral Telencephalic area, rostral portion (Vd_r_) and caudal portion (Vd_c_) of the dorsal nucleus of Ventral Telencephalic area, suppracomissural nucleus of Ventral Telencephalic area (Vs), central nucleus of Ventral Telencephalic area (Vc), medial (PPar_m_) and lateral (PPa_l_) portion of the anterior part of parvocellular preoptic area, posterior part of parvocellular preoptic area (PPp), magnocellular preoptic area (PM), ventral zone of periventricular hypothalamus (Hv), anterior tuberal nucleus (aTn) and periventricular nucleus of the posterior tuberculum (TPp). Dorsal and ventral habenula (Ha_d_ and Ha_v_, respectively) were also included in the analysis because, even if the habenular region is not reported as being part of the SDMN, it comprises a dual control system for conflict resolution of social aggression (Chou et al., 2016; Nakajo et al., 2020). Target brain areas are shown in Supplementary Figure 1, while abbreviations and putative homologies with mammals are summarized in Table 1.

**Table 1:** Summary of abbreviations of the teleost brain areas analyzed, with the corresponding putative homologies with the mammalian brain.

Abbreviations	Teleost brain region	Mammalian putative brain homolog
Dm	Medial zone Dorsal Telencephalic area	Basolateral Amygdala
Dl	Lateral zone of Dorsal Telencephalic area	Hippocampus
Vv	Ventral nucleus of Ventral Telencephalic area	Lateral Septum
Vd	Dorsal nucleus of Ventral Telencephalic area	Nucleus Accumbens; Striatum
Vc	Central nucleus of Ventral Telencephalic area	Nucleus Accumbens; Striatum
Vs	Suppracomissural nucleus of Ventral Telencephalic area	Medial amygdala and Bed nucleus of the stria terminalis
Ppa	Anterior part of the Parvocellular preoptic area	Preoptic area
Ppp	Posterior part of the Parvocellular preoptic area	Preoptic area
PM	Magnocellular preoptic area	Preoptic area
aTn	Anterior Tuberal nucleus	Ventromedial Hypothalamus
Hv	Ventral zone of periventricular hypothalamus	Anterior Hypothalamus
TPp	Periventricular nucleus of the posterior tuberculum	Ventral tegmental area
Had	Dorsal habenula	Medial habenula
Hav	Ventral habenula	Lateral habenula

*O’Connell and Hofmann (2011)*.

### Immunofluorescence for pS6

Brain activation was assessed by immunofluorescence for the neural activation marker phospho-S6 ribosomal protein (Knight et al., 2012). For antigen recovery, slides were treated with Tris-EDTA-Tween 20 (0.05%, pH 9.0) at 95°C for 20 min. Non-specific binding was blocked by incubating slides in TBS containing 1% bovine serum albumin (BSA) for 1 h prior to incubation in pS6 primary antibody (Cell Signaling pS6 ribosomal protein Ser235/236 antibody D57.2.2E Rabbit mAB #4858, 1:400 prepared in blocking solution) overnight at 4°C. Slides were rinsed in TBS-Triton X-100, incubated in secondary antibody (Alexa 594 Invitrogen goat anti-rabbit # A-11037, 1:1,000 prepared in blocking solution) for 2 h at RT, rinsed in TBS-Triton X-100, and finally mounted with VectaShield HardSet antifade mounting medium with DAPI (Vector Laboratories). Immunofluorescence staining was performed in brains from randomly selected animals per condition (male winners, male losers, isolated males, female winners, female losers, isolated females), with sample size per experimental condition varying between six and nine due to technical problems.

### Microscopy and Image Analysis

Brain sections were examined using Zeiss Axioscan. Z1 slide scanner and analyzed using the Zeiss Zen blue 2.1 imaging software. Images were acquired at 20× in .czi format and subsequently exported to Fiji software (Schindelin et al., 2012) for conversion to .tiff and .png format for panels arrangements. Quantification of immunoreactive pS6 cells was performed blind to the experimental conditions. In each section, positive pS6 cells were counted in a rectangle of 1,000 μm^2^ using the Zeiss Zen blue 2.1 imaging software within brain areas of interest (namely: Dm_m_, Dm_l_, Dm, Dl, Vv_r_, Vv_c_, Vd_r_, Vd_c_, Vs, Vc, PPa_m_, PPa_l_, PPp, PM, Hv, aTn, TPp, Ha_v_, Ha_d_). Within each brain area, quantification was performed in each of five consecutive coronal sections, and values were summed across these five sections to obtain individual data for each brain region.

### Statistical Analyses

Differences in latency, time of resolution, and frequencies of aggressive and submissive displays in males and females were expressed as means ± SE and compared by the *t*-test or Mann-Whitney in those cases in which data did not meet normality when assessed by the Shapiro-Wilks test. Two-tailed tests were used throughout the analysis. A *p*-value ≤0.05 was used as the threshold for significant difference. A distance-based permutation multivariate analysis of variance (perMANOVA, Anderson, 2001a; Anderson and Walsh, 2013) was performed to test differences in social behavior between males and females. Principal Component Analysis (PCA) was employed to visualize variability and explore variations in different behavioral displays in males and females. perMANOVA test was performed on Euclidean distance matrices on behavioral displays, with 999 random permutations (Anderson, 2001b). Total numbers of all behavioral displays were analyzed, and data were transformed by square root to correct for dispersion. For this multivariate analysis, behavioral displays including bites, chases, strikes, antiparallel displays, circles, flee, freeze, and retreat (Oliveira et al., 2011), were expressed as the total number of displays until conflict resolution. Statistical analysis corresponding to behavioral studies was performed using the computer program R (v 3.6.1), including packages vegan (2.5–6; Oksanen et al., 2019), RVAideMemoire (0.9–74; Hervé, 2020), factoextra (1.0.6; Kassambara and Mundt, 2019), ggcorrplot (0.1.3; Kassambara, 2019). Differences in pS6 immunostaining among social groups in each sex were analyzed by Kruskal-Wallis (KW), followed by false discovery rate multiple comparisons to correct for multiple brain areas and pairwise comparisons among ranks as *post hoc* test.

Weighted functional connectivity networks were constructed using the same boostrapping technique used in Nunes et al. (2021). In particular, multiple correlation network instances were constructed using all subsamples of size *s* = 7 of the population. Robustness analysis was conducted to confirm that results are robust to larger values of s. Each of the network instances was then thresholded using a density threshold of rho = 0.23, keeping the edges with the strongest weights (in absolute values, hence both positive and negative). The density was chosen based on the analysis of the estimated variance across the bootstrapped instances, and also coincided with the prescription of De Vico Fallani et al. (2017). The thresholded network instances were aggregated by averaging to obtain a single weighted network per treatment. These resulting treatment-specific networks were then separated in a positive edge weight and a negative edge weight network to separate the effects of positive coactivation (denoted excitation in the main text) and negative coactivation (denoted inhibition in the main text). For positive/negative edge weight networks, we tested whether the distributions were different across conditions (using both Kolmogorov-Smirnov test, KS, and Mann-Whitney’s *U* test, MWU). Finally, we computed eigencentrality rankings for each condition using all edges but weighted with the absolute value of their edge weight in order to preserve the positivity of eigencentrality values. Rankings of the nodes for the various conditions were listed and the nodes that are common between males and females among top-8 nodes in the eigencentrality ranking were extracted.

## Results

### Sex Differences in Agonistic Behavior and Temporal Dynamics of the Fights

Sex differences in aggressive behavior were assessed by quantifying the latency to the first attack, fight resolution time, and frequencies of behavioral displays. While there were no sex differences in the latency to the first attack (*W* = 435, *p* = 0.1195; Figure 1B), the resolution time was significantly lower in females (*W* = 242.5, *p* = 0.0015; Figure 1B) when compared to males. As a general pattern, in both sexes aggressive behavior included strike, bite and chase, while submissive behavior included flee, freeze and retreat. Moreover, the frequencies of aggressive and submissive displays were significantly higher in males than in females (*T* = 2.74, *p* = 0.0095; *T* = 2.33, *p* = 0.0256; Figure 1B). Moreover, when analyzing specific aggressive and submissive behaviors, females showed a significantly higher frequency of antiparallel displays than males (*T* = −3.13, *p* = 0.0035), while strikes and retreats showed the opposite trend (*T* = 2.5, *p* = 0.0174, *T* = 2.5; *p* = 0.0173; Figure 1B).

When analyzing contest dynamics, despite the fact that there was a large variability in duration and frequencies of behaviors in each encounter, as a general pattern in both sexes both opponents presented mutual assessment behaviors, such as antiparallel displays, circles, bites, and strikes (Figure 1C). Even if circles and antiparallel displays were observed throughout the entire encounter, usually bites, strikes, retreats, and freeze increases in frequency during the conflict. After the conflict resolution, there was a marked increase of other behaviors such as chases and flees, and all aggressive behaviors were initiated by the winner, while the loser displayed submissive behaviors.

To further explore how agonistic behavior can be reduced to major components that allow the discrimination between the sexes, we performed a Principal Component Analysis (PCA) with all measurements of agonistic behavior. The first and second principal components of the PCA explained 85.3% of total variation (PC1 and PC2 explaining 75.05% and 10.23%, respectively). PC1 was mainly associated with bite, strike and retreat, while PC2 was greatly explained by bite, chase, and flee (Table 2). Using perMANOVA to partition the euclidean distance by sex we found a significant difference in principal component loadings between males and females (*F* = 5.5502, *p*-value = 0.012; Figure 1D). Moreover, plotting PC1 scores of males and females also shows a significant sex-difference (MWU: *W* = 463.5, *p* = 0.0190; Figure 1E). These results suggest that there are differences in aggressive and submissive behavior between the sexes and that clustering into these two groups can be explained by the main components of agonistic behavior.

**Table 2:** Summary of principal components analysis (PCA) on behavioral traits.

	PC1	PC2
Bite	**−0.40023**	**−0.68837**
Antiparallel Display	−0.15065	0.03361
Circle	−0.21761	−0.27173
Chase	−0.11797	**0.43401**
Flee	−0.11857	**0.43537**
Strike	**−0.60677**	0.19781
Retreat	**−0.59203**	0.15410
Freeze	−0.15206	0.10191
Standard deviation	9.2649	3.4241
Proportion of variance	0.7505	0.1023
Cumulative proportion	0.7505	0.8530

*Heavy loadings of each component are shown in bold. The proportion of variance indicates the percentage of variation in the observed behavioral variables explained by each principal component (PC). Cumulative proportion indicates the sum total percentage of variation in the observed behavioral variables explained by a given PC and its preceding PCs*.

### Social Experience During Agonistic Encounters Activates Brain Regions Across the SDMN

**Figure 2:**
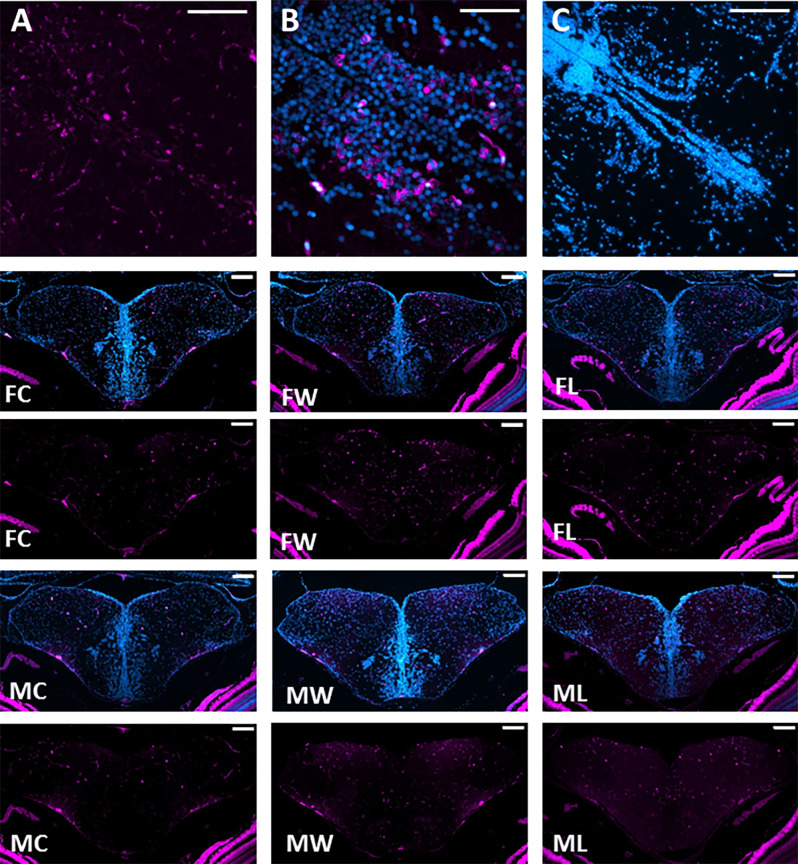
Neuronal activation throughout the brain Social Decision Making Network. **(A)** pS6 immunopositive staining with cytoplasmatic location is indicated in magenta. **(B)** Sections were co-stained with DAPI, indicated in cyan. **(C)** Negative control for pS6 and DAPI immunofluorescence. Representative photomicrographs of double DAPI (blue) and pS6 (magenta) and only pS6 staining (magenta) in Dm, Dl, Vc, Vd, and Vv of female controls (FC), female winners (FW), female losers (FL), male controls (MC), male winners (MW), and male losers (ML). Scale bars represent 100 mm.

**Figure 3:**
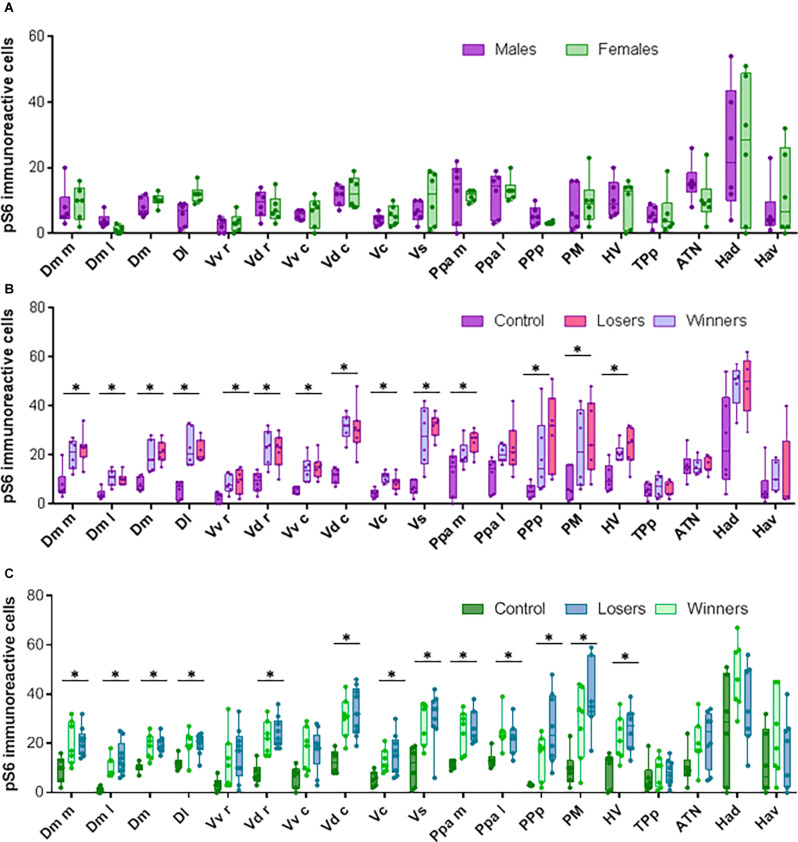
Neural activation in brain areas from the Social decision Making Network after aggressive encounters. **(A)** pS6 immunoreactive cells in brains from control animals that were not exposed to social interactions. **(B)** pS6 immunoreactive cells in males. **(C)** pS6 immunoreactive cells in females. Box plots were used to plot the data: the box extends to the furthest data points within the 25th and 75th percentile, and whiskers extend to the furthest data points not considered outliers. Asterisks indicate significant differences (*p* < 0.05) between controls and fish exposed to social interactions using the Kruskal-Wallis test.

Non-interacting male and female fish showed no differences in the number of pS6 immunoreactive cells in any of the brain areas analyzed (Figures 2, 3A). However, even if there were no differences between winners and losers in either sex, there was a higher number of pS6 positive cells in males and females exposed to an agonistic encounter when compared to isolated fish in most of brain areas (Dm_m_, Dm_l_, Dm, Dl, Vd_r_, Vd_c_, Vc, Vs, Ppa_m_, PP_p_, PM, HV; in the case of females, also PPa_l_, and in males also Vv_r_, and Vv_c_; Figures 2, 3B,C).

### Sex-Specific Functional Connectivity in the SDMN Changes in Response to an Agonistic Encounter

**Figure 4:**
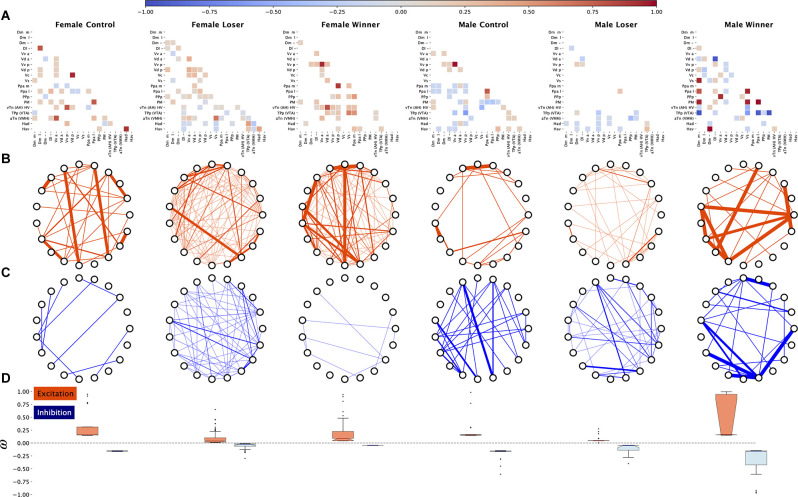
Functional connectivity across the brain regions induced by aggressive interactions, inferred from pS6 immunoreactive cells used as a marker of neural activity. **(A)** Adjacency matrices for each treatment showing the strongest negative and positive edges, sparsified for visualization puroposes, **(B)** excitation (positive weights) subnetworks for each treatment, **(C)** inhibition (negative weights) subnetworks for each treatment, **(D)** distributions of edge weights for excitation and inhibition subnetworks for each treatment.

Weighted functional connectivity networks were constructed for each experimental condition on each sex using bootstrapping (see “Materials and Methods” section for details), based on co-activation (i.e., correlation) matrices of the brain regions of the SDMN (Figures 4A–C).

A visual inspection of the adjacency matrices resulting from bootstrapping suggests sex differences in the predominance of positive (i.e., functional co-activation between brain regions) and negative (i.e., functional co-inhibition between brain regions) correlations among brain areas in all social treatments, with females showing mainly positive correlations (Figure 4A). Therefore, we decided to analyze the balance between excitation and inhibition in the SDMN between the sexes across the three experimental conditions (Figures 4B,C). There are significant differences between male and female controls in average excitation (MWU *s* = 1,508.0 *p* = 0.04) with an intermediate size effect (Cohen’s *d* = 0.29), no differences in Winners, and a significantly different distribution in Losers (KS *s* = 0.4686 *p* < 0.0001), with a larger variance for females (Figure 4B). Regarding inhibition, there were no significant sex differences in Controls, but there were significant differences both in distribution shape and mean both in losers (distribution: KS *s* = 0.58, *p* < 0.0001; median: MWU *s* = 5,416.0, *p* < 0.0001) and winners (distribution KS *s* = 1.0, *p* < 0.0001; median: MWU *s* = 828.0, *p* < 0.0001) with a large effect size (*d* = 0.68) in Losers a very large effect size in winners (*d* = 1.1; Figure 4C). Thus, in both Losers and Winners, this indicates a much stronger inhibition (larger negative values) in males as compared to females.

Changes in SDMN excitation-inhibition balance were also studied as a result of the fights in both females and males.

In females there were significant distribution and mean differences in excitation between Controls and Losers (KS *s* = 0.8, *p* < 0.0001; MWU *s* = 8,880.0, *p* < 0.0001, *d* = 1.3), and between Controls and Winners (KS *s* = 0.65, *p* < 0.0001; MWU *s* = 6,496.0, *p* < 0.0001, *d* = 0.7) with large effect sizes in both cases, pointing to a much larger excitation in Controls as compared with the other two conditions (Figure 4B). There were also significant differences between Losers and Winners (KS *s* = 0.52, *p* < 0.0001; MWU *s* = 8,104.0, *p* < 0.0001, *d* = −0.45) with an intermediate effect size, pointing to a larger excitation profile in Winners than Losers (Figure 4B). Inhibition in females was also significantly different in distribution shape and mean between Control and Losers (KS *s* = 0.88, *p* < 0.0001; MWU *s* = 150.0, *p* < 0.0001, *d* = −1.9) and between Controls and Winners (KS *s* = 1.0, *p* < 0.0001; MWU *s* = 0.0, *p* < 0.0001, *d* = −23.9) with very large effect sizes, pointing to much larger inhibition overall in Controls as compared with the other two conditions (Figure 4C). Differences between female Winners and Losers only occur in the distribution shape (KS *s* = 0.58, *p* < 0.0001; Figure 4C).

In males there were significant differences in distribution shape and mean excitation in all comparisons (Control vs. Losers: KS *s* = 0.912, *p* < 0.0001; MWU *s* = 2,652.0, *p* < 0.0001, *d* = 1.24; Control vs. Winners: KS *s* = 0.42, *p* < 0.0001; MWU *s* = 452.0, *p* < 0.001, *d* = −0.74; and Losers vs. Winners: KS *s* = 0.91, *p* < 0.0001; MWU *s* = 144.0, *p* < 0.0001, −1.7), implying larger excitation in Controls with respect to Losers, but also a smaller excitation in Controls with respect to Winners (Figure 4B). Regarding inhibition there were significant differences between Controls and Losers in distribution shape and mean (KS *s* = 0.8, *p* < 0.0001; MWU *s* = 572.0 *p* < 0.0001, *d* = −1.1) and between Winners and Losers (KS *s* = 0.8, *p* < 0.0001; MWU *s* = 2,768.0, *p* < 0.0001, *d* = 1.1457270256995857), with large effect sizes, pointing overall to a larger inhibition in Controls and Winners vs. Losers, but no significant difference between Controls and Winners, which implies that inhibition is reduced only in Losers (Figure 4C).

Finally, the balance between inhibition and excitation was compared for each condition in each sex (Figure 4D). An unbalance in excitation-inhibition was only found in female Winners (KS *s* = 0.52, *p* < 0.0001; MWU *s* = 1,868.0, *p* < 0.01, *d* = 0.63) and male Losers (KS *s* = 0.34, *p* < 0.001; MWU *s* = 1,656.0, *p* = 0.002, *d* = −0.55) with intermediate effect sizes, indicating that in female Winners excitation overcomes inhibition, while in male Losers inhibition overcomes excitation (Figure 4D).

**Figure 5:**
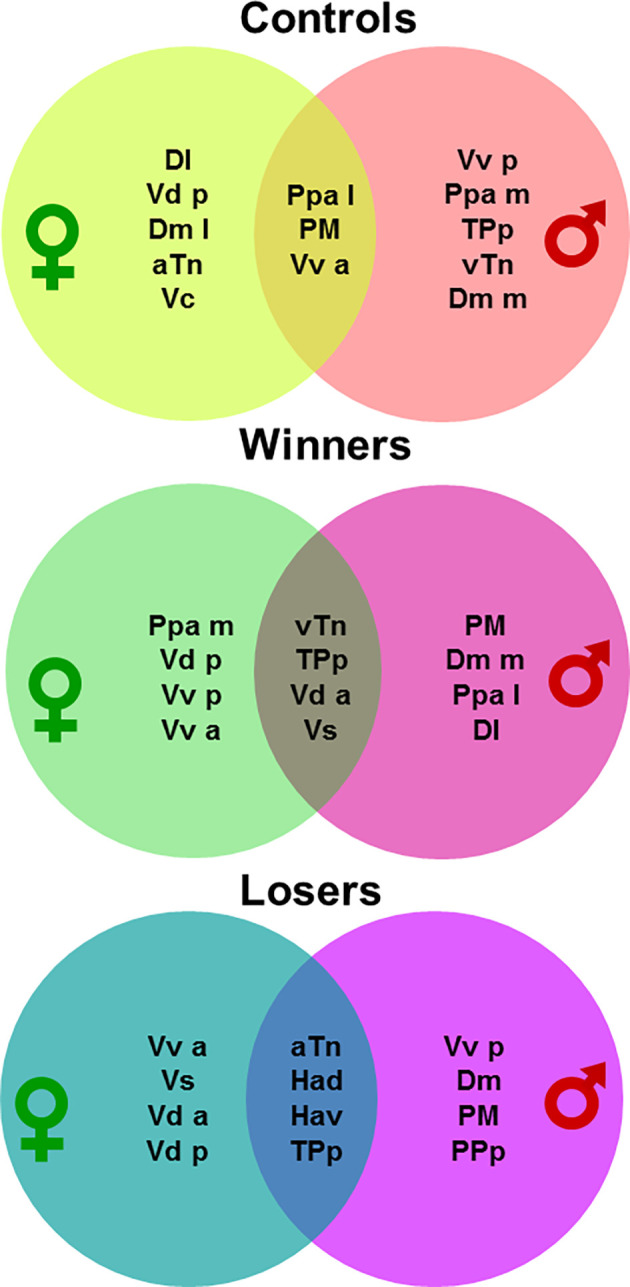
Venn diagrams representing the most central nodes of the brain social decision-making network in males and females for each social treatment (i.e., non-interacting control, winners, losers). For brain region abbreviations please see the “Materials and Methods” section.

The SDMN for each experimental treatment was also characterized using centrality network analysis. Using eigencentrality computed on absolute values of edge weights we have ranked the nodes from the highest (i.e., nodes connected to many nodes who themselves have high scores) to the lowest scores on each SDMN network, and we have arbitrarily selected the top 8 nodes on each condition to identify the most influential nodes in each network (Supplementary Table 1). The intersection of the top-8 regions for eigencentrality for each condition between the sexes varied between 3 out of 8 in Controls (PM, Vv a, Ppa l), to 4 out of 8 in both Winners (Vd a, Vs, TPp, Hv), and Losers (Hav, TPp, aTn, Had; Figure 5). These results indicate that central nodes in the SDMN network vary between males and females for each social condition, suggesting the occurrence of sex-specific SDMN network responses to agonistic interactions.

## Discussion

In the present study, we assessed the occurrence of sex differences in aggressive behavior and in the associated patterns of activation of the SDMN. Our results add insights into sex differences in agonistic behavior and on the neuronal architecture of intrasexual aggression in zebrafish. We showed that even if both sexes do not differ in the latency to the first attack and showed similar contest dynamics, they differed in fight resolution time and in the overall expression of agonistic behaviors. Males showed higher frequencies of aggressive and submissive behavioral patterns, while females presented more antiparallel displays and solved social conflict in less time. Aggression has been widely described in male intrasexual competitions but, despite the fact that females from different taxa also display complex aggressive behavior (Oliveira and Almada, 1996; Elekonich and Wingfield, 2000; Langmore et al., 2002; Davis and Marler, 2003; Borg et al., 2012; Renn et al., 2012; Scaia et al., 2018a, 2020), female aggression is still surprisingly understudied when compared to males. In our study, both female and male zebrafish showed high levels of intrasexual aggression, with females expressing the same agonistic behavioral repertoire as males during staged same-sex fights, as well as a similar temporal structure to the already described for male dyads of this species (Oliveira et al., 2011). Individuals from both sexes experienced a first phase showing mutual assessment behaviors, in which each animal determines the opponent’s relative fighting ability, and a second phase, in which aggressive behaviors were only initiated by the winner while the loser adopted submissive behaviors in response to the attacks. Latency to start the fight was similar between males and females, whereas the time of resolution was shorter in females than in males. The fact that both male and female zebrafish show a similar latency to initiate a fight when exposed to another same sex individual, even in the absence of limited resources to promote competition, such as a potential mate, shelter, or food, suggest that both sexes share a similar level of aggressive motivation. However, females show reduced time of conflict resolution and thus shorter fight duration, lower frequency of overt aggression behavioral patterns (i.e., strikes), and a higher frequency of signaling display (i.e., antiparallel display). The successful discrimination between males and females based on the principal components extracted from the agonistic behaviors PCA, further supports the occurrence of a sex-difference in fighting behavior. These results are in agreement with a previous study on dominance hierarchies in mixed-sex shoals of zebrafish suggesting that dominant females are less aggressive toward their subordinates than dominant males to their subordinates (Paull et al., 2010). Therefore, aggression in zebrafish seems to have been mainly selected within the scope of male intrasexual competition, being also present in females but with a reduction of putative maladaptive effects namely by a reduction in fight length and by the preferential use of antiparallel displays instead of overt aggression.

The use of pS6 as a marker of neuronal activation indicated that there were no sex differences in the activity of any of the studied brain regions in socially isolated individuals, and that agonistic interactions induced an increase in neuronal activity across most brain regions in both sexes (all but Tp_p_, Ha_d_ and Ha_v_ for both sexes, and also Vv_r_ and Vv_c_ for females).

Moreover, brain functional connectivity, as measured by the pattern of co-activation across brain regions of the SDMN, shows specific patterns of co-activation of the SDMN nodes for winners and losers both in males and females. The dynamics of the excitation and inhibition subnetwork responses to the fighting interaction, as assessed by using non-interacting control fish as a reference group with which to compare Winners and Losers, is different between the sexes, in particular for Winners. Winner males show increased excitation and no changes in inhibition, whereas Winner females show a decrease in both excitation and inhibition. For losers, both males and females show a decrease in both excitation and inhibition, with a higher decrease in inhibition in females. Moreover, the comparison of excitation and inhibition subnetworks between males and females in the same social condition shows that male Losers and Winners exhibit a stronger inhibition as compared to female Losers and Winners, respectively. Finally, when analyzing the balance between excitation and inhibition for each sex on each social condition, Winner females show an unbalance towards excitation while male Losers show an unbalance towards inhibition. In summary, the present data suggest sex differences in excitation-inhibition regulation in response to agonistic interactions and social status acquisition. Considering that changes in neural activity at nodes of the SDMN are related to the social environment and modulated by different molecules, including steroid hormones, neuropeptide hormones, neurotransmitters, and catecholamines (reviewed by O’Connell and Hofmann, 2012), these differences in excitation-inhibition could be related to differential modulation of the SDMN in both sexes. Neurological pathways regulating aggression in fish include different functional pathways that are also conserved in mammals such as dopamine, serotonin, histamine, nitric oxide, somatostatin, hypothalamo-neurohypophysial stem, hypothalamo-pituitary-interrenal and hypothalamo-pituitary-gonadal pathways, being arginine vasotocin, isotocin, dopamine, and serotonin key modulators of this behavior (Filby et al., 2010; Freudenberg et al., 2016; Teles et al., 2016a; Carreño Gutiérrez et al., 2020; Reichmann et al., 2020). When probing for the main neurobiological determinants of aggression in male and female zebrafish, principal component analysis suggests that social rank is a greater determinant for gene expression than sex, suggesting that in zebrafish similar expression profiles may regulate aggression in both sexes (Filby et al., 2010). However, one should keep in mind that this evidence refers to gene expression in large brain areas (e.g., telencephalon, optic tectum, hypothalamus, and hindbrain) and not in specific and more homogeneous brain nuclei. Even if regional dissection of the brains of zebrafish has been proven to be an accurate and valuable approach, other techniques such as micropunching, laser-capture microdissection, and immunofluorescence could assess the expression of neuromodulators in different nodes throughout the SDMN in males and females and could help to disentangle why functional connectivity includes mainly negative correlations in males, but positive ones in females.

We have also shown that the most influential nodes in each network, as detected by centrality analysis, are different between male and female SDMNs for each social treatment (i.e., controls, winners, and losers; see Figure 5). For controls, PM, Vv_a_ and PPa_l_ have high centrality in both females and males, but in females the other top eight centrality nodes are Dl, Vd_p_, Dm_l_, aTn, and Vc, whereas in males the other most central nodes are Vv_p_, PPa_m_, TPp, Hv, and Dm_m_.

For winners, females and males share Vd_a_, Vs, Tp_p_ and Hv as top centrality nodes, but in addition each sex has other four sex-specific top nodes (Ppa_m_, Vd_p_, Vv_p_, and Vv_a_ for females and PM, Dm_m_, Pp_al_, and Dl for males). For losers, there are four shared top centrality nodes between females and males (Ha_d_, Ha_v_, Tpp and aTn), and other four that are specific for females (Vv_a_, Vs, Vd_a_, Vd_p_,) and males (Vv_p_, Dm, PM, PPp). Therefore, each social treatment seems to induce a SDMN state with core nodes shared by each sex (controls: PM, Vv_a_, PPa_l_; winners: Vd_a_, Vs, Tpp, Hv; losers: Ha_d_, Ha_v_, Tp_p_, aTn), and another set of sex-specific top nodes for each treatment.

The core nodes for controls are putative homologs of the pre-optic area (PM, Ppa_l_) and the lateral septum (Vv_a_) in mammals (O’Connell and Hofmann, 2011) suggesting activity in the SDMN in the absence of social stimuli that engages salience monitoring in the environment, through the lateral septum (Rizzi-Wise and Wang, 2021) linked to the regulation of internal states, through the pre-optic area.

The core nodes for Winners contain putative homologs of the medial amygdala (Vs) and of the anterior hypothalamus (Hv) in mammals. Interestingly, in teleosts (i.e., rainbow trout, *Oncorhynchus mykiss*) the Vs projects to several hypothalamic regions including Hv (Folgueira et al., 2004), and stimulation of Vs elicits aggression in male bluegill fish (*Lepomis macrochirus*, Demski and Knigge, 1971). Moreover, the anterior hypothalamus in mammals is known to integrate neural processes related to agonistic behavior and is part of the “hypothalamic attack area”, which is a set of hypothalamic regions (e.g., perifornicai, anterior, lateral, and ventromedial hypothalamus) that do not coincide with a classical subdivision of the hypothalamus, and that elicit an attack when stimulated (Kruk et al., 1983; Hashikawa et al., 2017). Furthermore, in rats, stimulation of the attack area is accompanied by an increase in activity in the medial amygdala (Halász et al., 2002), suggesting a joint involvement of the medial amygdala and the hypothalamic attack area in the regulation of aggression in both fish and mammals. The other two areas that are shared between the sexes in winners are the Tp_p_ and the Vd_a_, which are putative homologs of the mammalian ventral tegmental area and nucleus accumbens, respectively (O’Connell and Hofmann, 2011). The centrality in winners SDMN’s of these two regions, that are part of the dopaminergic mesolimbic system, may be related to a rewarding value of winning a social interaction. Apart from these shared central nodes between the sexes, male winners also have as central nodes homologs of the mammalian pre-optic area (PM, Ppal), amygdala (Dm_m_) and hippocampus (Dl), whereas females have homologs of the pre-optic area (Ppa_m_), nucleus accumbens (Vd_p_) and lateral septum (Vv_a_ and Vv_p_). Therefore, the main sex differences in the winners’ SDMN hubs are involvement of hippocampus and central amygdala homologs in males and of lateral septum homolog in females.

The core nodes for Losers contain putative homologs of the habenula, the ventral tegmental area, and the ventromedial hypothalamus. The teleost dorsal habenula and its homolog medial habenula in mammals have been involved in dominance behaviors and innate fear response, whereas the teleost ventral habenula and its homolog lateral habenula in mammals have been implicated in learning active avoidance (Okamoto et al., 2021). Therefore, the centrality of the habenula in the Losers SDMN’s may reflect fear and avoidance associated with a losing experience. In line with this suggestion, the centrality of the ventral tegmental area may be related to signaling the aversive valence of the interaction, since this region is known to process not only rewarding but also aversive stimuli, with specific neuronal populations processing different valences (e.g., Lammel et al., 2012; Tan et al., 2012), and its projections to the lateral habenula have been shown to be involved in aversive conditioning (Root et al., 2014). Finally, the centrality of the ventromedial hypothalamus, which is also part of the hypothalamic attack area, and recently has been shown to contain a more restricted ventrolateral part that is a key area for agonistic behaviors (Lin et al., 2011), may reflect the expression of aggression also by losers during the agonistic encounter. Apart from these shared central nodes between the sexes, male losers also have as central nodes homologs of the mammalian pre-optic area (PM, Pp_p_), amygdala (Dm), and lateral septum (Vv_p_), whereas females have homologs of the medial amygdala (Vs), nucleus accumbens (Vd_a_, Vd_p_) and lateral septum (Vv_a_). Therefore, the main sex differences in the losers SDMN hubs are an involvement of pre-optic area and central amygdala homologs in males and of medial amygdala and nucleus accumbens homologs in females.

It is worth mentioning that overall the central nodes of the SDMN identified here correspond to homolog areas identified in mammalian brains has key brain regions in the regulation of aggression, namely the anterior hypothalamus (Hv), basolateral amygdala (Dm), medial amygdala (Vs), preoptic area (PM, Pp_a_, Pp_p_), and the ventromedial hypothalamus (aTn; for a review of proposed homologies see O’Connell and Hofmann, 2011). Together, these results suggest that the state of the SDMN, as captured by the pattern of co-activation of its nodes, is already different between males and females that are not engaged in social interactions, and that the SDMN responds to experiencing a win or a defeat in a sex-specific manner. Moreover, these results also show that the behavioral differences in male and female aggression are better reflected by the pattern of co-activation of the brain regions that constitute the SDMN, rather than by the activity of each of the brain regions *per se*.

Previous studies using neuronal markers to characterize the association between neuronal activity and aggression in fish, have mainly focused on the activity of single nodes of the SDMN and not on its pattern of functional connectivity (*A. burtoni*: Burmeister et al., 2005; Loveland and Fernald, 2017; *Mchenga conophoros* and *Petrotilapia chitimba*: Baran and Streelman, 2020). In most of these studies differences in neuronal activation have been described in specific brain regions. For example, in the cichlid fish *Astatotilapia burtoni*, males descending in social status (i.e., losers) presented a higher neuronal activation of different brain areas, such as Dm, Vv, Vs, Vd, and ATn, than non-descending animals (e.g., winners) when using the expression of immediate early genes (*cfos*, *egr1*; Maruska et al., 2013) or pS6 (Butler et al., 2018) as markers of neuronal activation. However, it is worth mentioning that patterns of brain activation vary if social defeat is assessed after one defeat or after repeated subsequent defeats (Martinez et al., 1998; Butler et al., 2018). This could explain the differences in patterns of brain activation between the current study (where despite an increase in neuronal activity across most areas of the SDMN after social interaction, no differences were observed between winners and loser for both sexes) and those presented in a previous study in zebrafish, in which losers and winners presented differences in the expression of immediate early genes in different areas of the SDMN (Teles et al., 2016a). While in the present study agonistic encounters were stopped immediately after conflict resolution in order to assess functional connectivity after winning or losing, in the former case encounters lasted for 30 min. Thus, depending on how long each conflict resolution took, different levels of expression in immediate early genes could reflect the recently acquired social status.

Fewer studies have characterized the patterns of functional connectivity based on neuronal markers, and those that have done so only assessed one of the sexes. For example, Teles et al. (2016a) reported different patterns of functional connectivity across a subset of nodes of the SDMN between winners and losers of agonistic encounters in male zebrafish, and Butler et al. (2018) identified differences in functional connectivity of the SDMN between males of the cichlid fish *Astatotilapia burtoni* that respond to repeated social defeat in either a proactive or a reactive fashion. The functional connectivity of the SDMN in females has also been studied in cichlids, but in relation to mouthbrooding behavior (Maruska et al., 2020). Thus, to the best of our knowledge, the present study provides the first comparison of brain states between the sexes when performing aggressive behavior in the same ethological context. Our results indicate a distinct neural activation pattern associated with social experience during fights, suggesting that social defeat reduces functional connectivity (both excitatory and inhibitory) throughout the SDMN regardless of the sex. In this sense, evidence on male brown anole (*Anolis sagrei*) suggests that functional connectivity within the SDMN is decreased as a consequence of an agonistic challenge, even if opponents were not classified as winners or losers (Kabelik et al., 2018). Taking this into account, this study further supports previous evidence that already suggested that neural activity across the SDMN nodes varies across social conditions (Teles et al., 2016a; Kabelik et al., 2018).

Overall, our study adds insights into the neural basis underlying sex differences in agonistic behavior in a species in which both females and males display complex aggressive behavior. This study presents the first experimental evidence showing that functional connectivity is modified after an acute agonistic interaction in both sexes, with sex differences in the brain excitatory-inhibitory balance of the SDMN. Considering that the most influential nodes in each network are different across social treatments in a sex-specific manner, these results support a sex-specific differential activation of the social brain as a consequence of social experience. These sex differences observed in the pattern of activity of the SDMN that parallels the expression of sex-specific patterns of agonistic behavior in zebrafish, supports the hypothesis of the coevolution of constraints in the mechanisms underlying aggression between the sexes, this way reducing the maladaptive consequences of aggression for females in a scenario in which aggression is mainly selected for in males.

## Data Availability Statement

The raw data supporting the conclusions of this article will be made available by the authors, without undue reservation.

## Ethics Statement

The animal study was reviewed and approved by DGAV (Direção Geral de Alimentação e Veterinária, Portugal).

## Author Contributions

MS and RO contributed to the conception and design of the study. MS ran the behavioral experiments. MS and IA ran the immunocytochemistry assays. MS acquired all data and curated the database. MS and IA performed the statistical analysis. GP developed the pipeline for the network analysis and ran the analysis. MS and RO wrote the first draft of the manuscript. All authors contributed to the article and approved the submitted version.

## Conflict of Interest

The authors declare that the research was conducted in the absence of any commercial or financial relationships that could be construed as a potential conflict of interest.

## Publisher’s Note

All claims expressed in this article are solely those of the authors and do not necessarily represent those of their affiliated organizations, or those of the publisher, the editors and the reviewers. Any product that may be evaluated in this article, or claim that may be made by its manufacturer, is not guaranteed or endorsed by the publisher.

## Figures and Tables

**Figure:**