# Supplementary material for: Sex Differences in Aggression Are Paralleled by Differential Activation of the Brain Social Decision-Making Network in Zebrafish
Source: Front Behav Neurosci. 2022 Feb 16;16:784835. doi: 10.3389/fnbeh.2022.784835 (PMC8890505; doi:10.3389/fnbeh.2022.784835)
Supplement: Supplementary file 1 [file Data_Sheet_1.pdf]

SUPPLEMENTARY MATERIAL

Scaia, M.F., Akinrinade, I., & Oliveira, R.F. (2021). Sex-differences in aggression are paralleled by differential activation of the brain social decision-making network in zebrafish. *Frontiers in Behavioral Neuroscience*.

SUPPLEMENTARY FIGURE

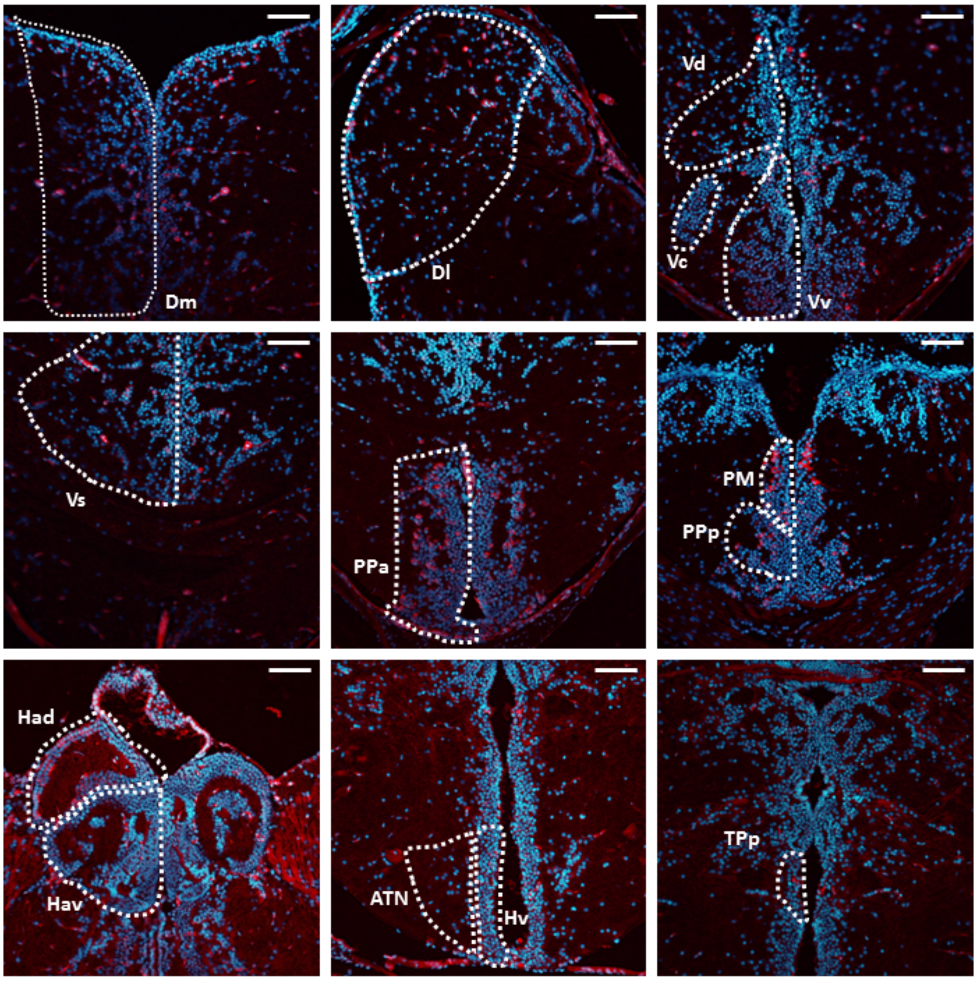

Figure S1: Location of brain regions from the SDMN in which brain activation was determined. Red staining indicates pS6 immunopositive cells, and blue staining correspond to DAPI. Abbreviations: ATN: anterior tuberal nucleus; Dl: lateral zone of the dorsal telencephalon; Dm: medial zone of the dorsal telencephalon; Had: dorsal habenula; Hav: ventral habenula ; Hv: ventral zone of periventricular hypothalamus; PPa: anterior part of the

parvocellular preoptic area; PPp: posterior part of the parvocellular preoptic area; PM: magnocellular preoptic area; TPp: periventricular nucleus of the posterior tuberculum; Vd: dorsal nucleus of the ventral telencephalon; Vc: central nucleus of the ventral telencephalon; Vs: supracommissural nucleus of the ventral telencephalon; Vv: ventral nucleus of the ventral telencephalon. Scale bars represent 100µm.

SUPPLEMENTARY TABLE

Table S1. Ranking of eigen centralities of each node of the SDMN for males and females of each social treatment.

Female Control	Female Loser	Female Winner	Male Control	Male Loser	Male Winner
Ppa l	aTn (VMH)	Ppa m	Vv p	Vv p	PM
PM	Had	Vd p	Vv a	TPp (VTA)	Dm m
Dl -	Vv a	vTn (AH) HV	Ppa m	Hav	Ppa l
Vd p	Vs	TPp (VTA)	Ppa l	Had	Vs
Dm l	Hav	Vd a	TPp (VTA)	Dm -	TPp (VTA)
aTn (VMH)	Vd a	Vv p	PM	aTn (VMH)	Dl -
Vc	Vd p	Vs	vTn (AH) HV	PM	Vd a
Vv a	TPp (VTA)	Vv a	Dm m	PPp	vTn (AH) HV
vTn (AH) HV	Vc	PPp	PPp	Vc	Hav
Vv p	Dl -	Hav	Dl -	Dl -	Dm -
Had	PPp	Dl -	Dm -	Ppa m	Vd p
Vd a	Vv p	aTn (VMH)	Hav	Vd a	PPp
Ppa m	Dm l	Had	Had	Vv a	Vv p
Hav	Ppa m	Dm -	Dm l	Dm m	Dm l
Dm m	Dm m	PM	Vd a	Ppa l	Vv a
Dm -	vTn (AH) HV	Ppa l	Vs	Vs	Had
PPp	Ppa l	Dm m	aTn (VMH)	Vd p	Vc
Vs	Dm -	Vc	Vd p	Dm l	aTn (VMH)
TPp (VTA)	PM	Dm l	Vc	vTn (AH) HV	Ppa m
